# Beyond the algorithm: why oncology nursing in Asia–Pacific needs evidence-based AI evaluation

**DOI:** 10.1016/j.apjon.2026.100939

**Published:** 2026-03-20

**Authors:** Karthik Adapa, Suguna Kotte, Mounika Metta, Stefan Escobar-Agreda, Resham Sethi, Girish Patil, Lukasz Mazur

**Affiliations:** a Department of Health Systems, World Health Organization-South East Asia Regional Office, New Delhi, India; b Division of Healthcare Engineering (DHE), Department of Radiation Oncology - School of Medicine, University of North Carolina, Chapel Hill, United States; c Koita Centre for Digital Health, Ashoka University, Sonipat, Haryana, India

A nurse working in a busy oncology ward in Manila receives an alert from a newly installed artificial intelligence (AI) clinical decision support system. The tool recommends adjusting the symptom management plan for a patient undergoing chemotherapy for gastric cancer. The algorithm's confidence score is high. But how does she know whether to trust it? Has this tool been validated on Filipino patients? Has anyone evaluated whether it improves the outcomes she cares about most, patient comfort, timely intervention, safe care transitions? These are not hypothetical questions. They represent the daily reality confronting oncology nurses across the Asia–Pacific as AI tools proliferate in cancer care settings.

We argue that the rapid adoption of AI in oncology across the Asia–Pacific region has dramatically outpaced the evidence base required to guide its responsible use. Oncology nurses must demand and lead rigorous, context-sensitive evaluation of these tools. Moving beyond technical accuracy to determine whether AI improves cancer care in diverse clinical environments of our region. Achieving this goal requires methodological innovation, drawing upon pragmatic trials, hybrid designs, real-world evidence (RWE), and health technology assessment (HTA) frameworks that can accommodate the unique characteristics of AI-based health interventions.

## What is AI evaluation?

AI evaluation is the systematic process of measuring the behavioural properties, clinical performance, and societal impact of AI systems to inform decisions about their development, deployment, and ongoing use in health care.^1^ This definition reflects a growing consensus that evaluation must extend well beyond the technical metrics that have historically dominated the field.

A useful distinction exists between three levels of assessment. Technical validation examines whether algorithms perform accurately on test data. Clinical evaluation determines whether these tools improve patient outcomes in practice. Real-world impact assessment considers broader issues such as safety, equity, workflow integration, cost, and effects on the clinician–patient relationship. All three levels are necessary; none alone is sufficient.^2^^,^^3^

**Fig. 1:**
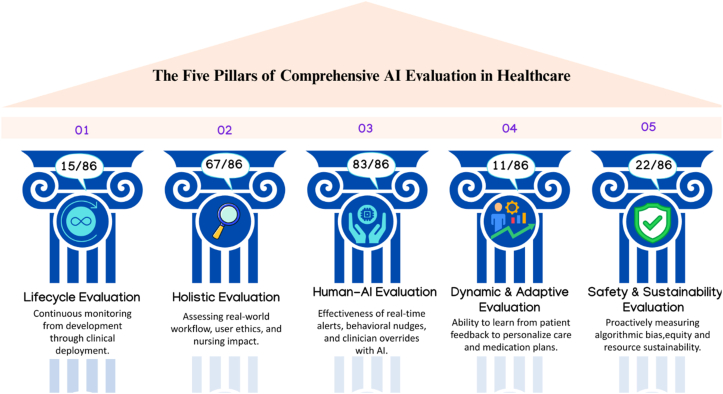
Five pillars of comprehensive AI Evaluation in health care. AI, artificial intelligence.

Fig. 1 presents a framework for the evaluation of AI within health care delivery categorized into five strategic pillars. The numerical values within the circular indicators represent the prevalence of these evaluation criteria across 86 identified clinical trials, highlighting current research priorities and gaps (Supplementary material). Five essential pillars for comprehensive evaluation have been proposed: lifecycle evaluation that continuously monitors AI from development through deployment; holistic evaluation that moves beyond benchmarks to assess real-world performance, user interactions, and ethical implications; human and automated evaluation that balances nuanced human judgement with scalable automated methods; dynamic and adaptive evaluation that recognises benchmarks can grow stale and must evolve alongside the technology; and safety and sustainability evaluation that proactively measures risk, bias, and environmental impact.^4^ For oncology nursing, this framework is invaluable because it insists that evaluation cannot be a one-time technical exercise, it must be ongoing, multidimensional, and anchored in how AI actually functions within clinical workflows.

The distinction between controlled laboratory evaluation and real-world evaluation is critical. Standardised benchmarks and datasets offer reproducible, controlled assessments of model capabilities, but they often fail to capture how AI performs amid the unpredictable, complex conditions of real clinical practice.^4^^,^^5^ The metrics that matter to a procurement officer or a frontline nurse, such as time saved, patient outcomes improved, and workflow disruption minimized, may bear little resemblance to benchmark accuracy scores. This gap between laboratory performance and real-world utility is precisely the space that oncology nursing must help fill.

Validation taxonomies from the systems engineering literature further clarify the landscape, identifying four primary approaches: trial-based validation with real users, simulation in synthetic environments, model-centred validation of performance metrics, and expert opinion.^5^ Each has a role at different stages of the AI lifecycle, and post-deployment continuous validation, through failure monitoring, safety channels, and ongoing performance tracking, is essential once tools enter clinical use. The Translational Evaluation of Health care AI (TEHAI) framework organises this progression around three domains: capability (does the model work?), utility (does it add clinical value?), and adoption (will clinicians and patients accept and use it?).^6^^,^^7^ All three must be rigorously assessed before any AI tool can be considered ready for oncology practice.

## Why AI evaluation matters

### The evidence gap is real and consequential

**Fig. 2:**
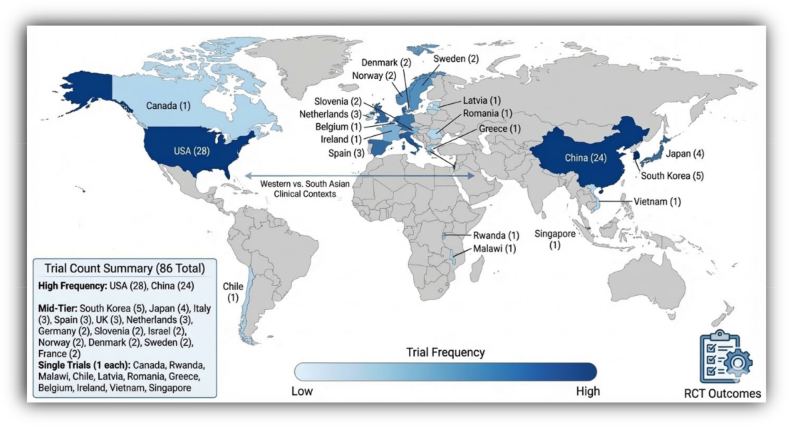
Global Distribution of randomized controlled trials for AI evaluation (adapted from Han et al.). AI, artificial intelligence.

The scale of the current evidence deficit is striking. A scoping review of randomised controlled trials (RCTs) evaluating AI in clinical practice found that of 86 such trials conducted worldwide between 2018 and 2023, only four took place in low- and middle-income countries.^8^ The vast majority of evidence on AI in health thus derives from settings that bear little resemblance to many Asia–Pacific contexts, where infrastructure varies enormously, workforce shortages are acute, and patient populations differ in genetics, disease burden, and cultural expectations of care. Fig. 2 depicts the global AI evidence gap among regions.

AI systems produce rich engagement data and strong technical metrics, but these alone do not show whether interventions improve real-world outcomes like health and well-being.^9^ Without rigorous impact evaluation, promising AI tools may stall between proof-of-concept and meaningful benefit, wasting resources and potentially widening care disparities. Standardized evaluation methods have lagged behind generative AI deployment, creating a gap between lab-tested performance and real-world impact.^4^

### Lessons from prior technology failures

History shows that seemingly transformative technologies like classroom laptops or improved cookstoves often fail when evaluation focuses on the tool rather than context, delivery, and human behavior.^9^ Oncology offers a cautionary example: IBM Watson for Oncology, deployed across major centers in India, South Korea, Thailand, and China, showed limited concordance with expert recommendations and poor contextual relevance, leading some institutions to discontinue use.^10^ This highlights that technical sophistication alone does not ensure clinical value and that the Asia–Pacific region has already faced the risks of adopting AI without proper evaluation.

### The stakes for Asia–Pacific oncology

The Asia–Pacific region accounts for roughly half of global cancer cases and deaths, with rapidly rising incidence, severe workforce shortages especially in nursing and wide urban–rural disparities.^11^ AI is being promoted for screening, diagnostic imaging, treatment support, and symptom management, with the regional AI-in-oncology market growing at an estimated 30.7% compound annual growth rate, outpacing evidence generation.^12^

Deploying unevaluated AI carries significant risks: algorithmic bias from data unrepresentative of Asian populations, diagnostic tools untested in resource-limited settings, erosion of clinical judgment through over-reliance on automation, and care processes designed without patient- or nurse-centered input. Evaluating downstream human impacts not just technical accuracy is critical.^4^^,^^13^ A false-negative screening result in a rural Philippine clinic carries profoundly different consequences than in a well-resourced urban hospital. Without context-sensitive evaluation, we cannot distinguish AI tools that help from those that harm.

### Evidence-based methods for conducting AI evaluation

A central challenge in AI evaluation is selecting study designs capable of generating evidence that informs clinical adoption, policy, and regulatory decisions. While RCTs remain the traditional gold standard for establishing causal effects, they are often insufficient for AI systems that evolve over time, interact with clinical workflows, and produce context-dependent outcomes.^14^^,^^15^ AI tools differ fundamentally from pharmaceuticals and static medical devices: they may update continuously, interact with complex clinical workflows, and produce effects that are highly context-dependent. This section examines the principal methodological approaches available for AI evaluation and their relative strengths. The principal evidence-based methods used for AI evaluation, along with their purpose, importance, examples, and key strengths, are summarised in Table 1.

**Table 1:** Evidence-based approaches for evaluating AI interventions.

Method	Purpose	Importance for AI Evaluation	Evidence	Key Strengths
Randomised controlled trials (RCTs)	Establish causal effects of AI interventions on patient outcomes.	Provides rigorous evidence to determine whether AI tools improve clinical care; highlights limitations of relying on technical performance alone.	Scoping review of 86 AI RCTs (2018–2023) showed most were single-centre with limited demographic reporting; nearly half did not improve patient outcomes despite positive technical performance.^7^^,^^16^	Strong internal validity; enables causal inference; well-established methodology.
Pragmatic trials	Evaluate AI effectiveness in routine clinical practice.	Captures real-world utility and integration into clinical workflows; informs decisions under practical conditions.	Stepped-wedge cluster randomised trials where units adopt AI sequentially; implementation trials that focus on uptake in practice.^17^^,^^18^	Focuses on patient-centered outcomes; adaptable to diverse settings; accommodates dynamic AI systems.
Hybrid Effectiveness–Implementation designs	Assess both clinical effectiveness and implementation strategies simultaneously.	Determines whether AI tools improve outcomes and whether they can be successfully integrated into care.	Type 1, 2, 3 hybrid designs; Type 2 particularly useful in oncology to evaluate both patient outcomes and workflow integration.^17^^,^^19^	Integrates research and practice; flexible design, adapts to evolving systems like LLM's; evaluates implementation success and clinical impact together.
Real-world evidence (RWE) / Real-world data (RWD)	Monitor AI performance using data from routine clinical practice.	Detects performance degradation, dataset shift, rare adverse events, and equity impacts that trials may miss.	Data from electronic health records, patient registries, claims databases, and patient-generated data.^20^^,^^21^	Continuous evaluation post-deployment; reflects heterogeneous clinical settings; essential for bridging prototype-to-bedside gap.
Health technology assessment (HTA)	Evaluate clinical, economic, organisational, and ethical implications of AI technologies.	Supports policy and adoption decisions; ensures equity, sustainability, and workflow impact are considered.	Delphi survey of 46 European experts identified 48 topics for AI-specific HTA, including patient safety, model accuracy, benefit-harm balance; Digi-HTA (Finland), NICE evidence standards, EUnetHTA adaptation.^22^, ^23^, ^24^, ^25^	Multidisciplinary evaluation; considers cost-effectiveness, organisational readiness, long-term sustainability; supports evidence-based policy.

AI, artificial intelligence; LLM, Large Language Model.

Together, these methods provide a complementary toolkit for evaluating AI interventions in oncology nursing. By combining traditional RCTs, pragmatic designs, hybrid trials, real-world evidence, and health technology assessment, researchers and clinicians can generate robust, context-sensitive evidence that informs safe, effective, and equitable AI adoption.

## Current state of AI evaluation in oncology

### The global landscape

Recognition of the AI evaluation gap is accelerating at the global level. In February 2026, the Gates Foundation, Novo Nordisk Foundation, and Wellcome launched the EVAH initiative with a joint investment of US$ 60 million to support locally led evaluations of AI health tools in low- and middle-income countries.^26^ This highlights the need for lifecycle evaluation, ensuring continuous monitoring from development through deployment, and holistic evaluation, capturing real-world performance, ethical considerations, and user interactions. The initiative mandates RCTs, implementation science studies, economic analyses, and assessments of professional and public trust.

Yet the field remains fragmented. Six distinct evaluation paradigms have been identified, including benchmarking, evals, construct-oriented assessment, exploratory evaluation, real-world impact studies, and testing/evaluation/verification/validation (TEVV), each developing along largely insular trajectories with conflicting terminologies and limited cross-pollination.^1^ On the regulatory front, the EU AI Act, US FDA framework for AI-based medical devices, and UK NICE Evidence Standards Framework represent important but uncoordinated efforts, and most lack specific provisions for Asia Pacific nursing-relevant outcomes.^26^

### The Asia–Pacific context

Within Asia–Pacific, AI adoption is advancing rapidly, but evaluation infrastructure lags far behind. A scoping review of AI in cancer screening across ASEAN reveals significant heterogeneity in both screening programme maturity and AI readiness.^27^ Country-level variations are stark: Japan and South Korea lead in AI development, but evaluation frameworks have not kept pace with adoption; India's ICMR ethical guidelines represent emerging but fragmented regulation; China has made massive AI investments without proportionate evaluation rigour; and Southeast Asian nations face compounding challenges of limited infrastructure, workforce constraints, and questions about data representativeness.^10^^,^^26^ These gaps emphasize safety and sustainability evaluation, proactively addressing risk, bias, and long-term impacts on patients and nursing workflows.

### Gaps in oncology nursing

Perhaps most critically, existing AI evaluation studies in oncology focus largely on physician-centred tasks such as diagnosis, imaging interpretation, and treatment selection. Far less attention has been given to outcomes central to oncology nursing practice, including symptom management, patient education, care coordination, psychosocial support, and survivorship care.^8^^,^^28^ Evidence is also lacking on how AI systems affect nursing workload, clinical judgement, professional autonomy, or the nurse–patient therapeutic relationship.^4^ Across the culturally and linguistically diverse health care systems of the Asia–Pacific region, this absence of nursing-focused evaluation represents a significant blind spot that must be addressed.

## Best practices for AI evaluation in oncology

### Articulate a theory of change

Three foundational decisions shape the risks and results of any AI programme: articulating a clear theory of change, assessing whether the programme can realistically be implemented, and planning for successful scale.^9^ In oncology, this means specifying how an AI tool improves outcomes through which pathway, for which patients, in which setting, and via which human actions. A six-pathway taxonomy improving targeting, increasing access to personalized support, maximizing frontline worker effectiveness, enhancing efficiency, reducing bias, and boosting resource mobilization offers a framework for defining these mechanisms^9^ Evaluations should also clearly state the tool's intended use, primary user, integration point in the care pathway, and expected clinical action, aligning outcomes with real-world tasks.^3^

### Design rigorous, context-appropriate evaluations

RCTs remain key for establishing causal impact, especially when AI interacts with complex behaviors, incentives, and workflows. They should be complemented by pragmatic trials, hybrid designs, real-world evidence (RWE), and health technology assessments (HTA).^14^^,^^17^^,^^29^ Evaluation must be tailored to specific, practical use cases, guided by three overarching principles: defining contextually appropriate performance metrics aligned with real-world stakeholder priorities; capturing unintended impacts using data representative of actual practice conditions; and considering workflow effects by examining how AI outputs interact with the full decision-making process.^4^^,^^16^ For oncology nursing, this means measuring whether accurate AI assessments change decisions and improve patient outcomes.

### Evaluate beyond technical performance

Evaluation must encompass performance, fairness, safety, ethical implications, energy and resource costs, and alignment with human values.^4^ Stakeholders including engineers, clinicians, policymakers, and patients offer distinct perspectives. Continuous evaluation is essential as benchmarks age, models evolve, clinical practices shift, and data leakage inflates apparent performance.^4^^,^^30^ For oncology nursing in Asia–Pacific, this means building evaluation protocols that incorporate capability, utility, and adoption domains,^7^ economic and acceptability assessments,^31^ and nursing-specific outcomes like workflow integration, professional satisfaction, autonomy, and nurse–patient relationship quality.

### Prioritise equity and open dissemination

Evaluation must prioritise tools trained on representative data, suitable for resource-constrained environments, and focused on public benefit. Evaluation must disaggregate performance across ethnicity, socioeconomic status, geography, gender, and age to detect disparities. Teams should include local researchers with contextual expertise.^9^^,^^26^ The fragmentation that characterises the current evaluation landscape can only be overcome through transparent dissemination of both positive and negative evaluation results.^1^

### Recommendations for Asia–Pacific oncology nursing

Drawing on the frameworks and evidence reviewed above, we offer six recommendations for advancing AI evaluation in oncology nursing across the Asia–Pacific.

First, establish an Asia–Pacific AI Evaluation Consortium for Oncology Nursing. Modelled on the EVAH partnership structure,^31^ this consortium should unite academic nursing institutions, cancer centres, and AI developers across the region's diverse income levels. Using harmonized yet context-specific protocols ensures evidence generated in one setting can inform regional decision-making.

Second, develop an oncology nursing-specific AI evaluation framework. Existing models must be adapted to include nursing-sensitive domains: patient safety, workflow integration, clinical judgment, and symptom management outcomes.^7^^,^^16^ This framework must be scalable across resource settings, equally useful for a tertiary cancer centre in Tokyo and a district hospital in rural Indonesia.^10^

Third, prioritise locally led, contextually adapted evaluations. Evaluations should be co-designed with local oncology nurses, patients, and communities, prioritizing high-burden regional cancers. Assessments must address linguistic and cultural appropriateness to mitigate the performance gaps of AI trained primarily on English-language data.^27^^,^^31^

Fourth, embed evaluation into AI deployment from the outset. When AI and evaluation should be integrated into core systems to guide scaling or adaptation decisions.^9^ Health systems across Asia–Pacific should mandate pre-deployment evaluation protocols before adopting AI tools in oncology, with ongoing post-deployment monitoring that tracks nursing-sensitive indicators and patient-reported outcomes. Continuous lifecycle evaluation recognising that AI models trained on historical data may degrade as clinical practices evolve reinforces the need for sustained monitoring well beyond initial deployment.^16^^,^^30^

Fifth, invest in evaluation capacity building. Train oncology nurses and researchers in AI literacy and implementation science. Essential investments include regional training programs, mentorship networks for early-career scientists, and the integration of AI evaluation competencies into nursing curricula.

Sixth, advocate for regional regulatory harmonisation. The current regulatory fragmentation across Asia–Pacific creates regulatory arbitrage and uneven patient protection. The oncology nursing community should engage actively with emerging ASEAN health technology assessment networks and World Health Organization (WHO) frameworks, ensuring that the nursing voice is present where AI evaluation standards are being set.^22^^,^^26^^,^^27^ Focusing on regulating outcomes and societal impacts rather than specific technologies offers a flexible, adaptable model well-suited to the region's diverse regulatory environments.

## Conclusions

Return now to that oncology nurse in Manila. She does not need another AI tool with an impressive accuracy score derived from a benchmark dataset of North American patients. She needs evidence—generated in settings like hers, evaluated against outcomes she cares about, tested with patients who resemble hers, and assessed for how it integrates into the workflows she navigates daily.

The Asia–Pacific oncology community stands at a pivotal juncture. AI adoption is accelerating across the region, driven by an immense cancer burden and workforce shortages, Yet the evaluation infrastructure has not kept pace. Current practice lacks a holistic, continuous, and dynamic approach to testing performance in real-world scenarios^4^ and a necessity in this region given diversity of health care contexts, patient populations, and resource levels makes context-sensitive evaluation not a luxury but a necessity.

Critically, the methodological toolkit for AI evaluation must expand beyond the conventional RCT. Pragmatic trials, hybrid designs, RWE generation, and HTA frameworks each address dimensions that traditional trial designs cannot.^14^^,^^17^^,^^22^^,^^29^^,^^30^ Principled integration of these approaches guided by causal roadmaps and implementation science will be essential for generating evidence that is both scientifically rigorous and clinically actionable.^21^

Ultimately, AI will not transform oncology care on its own. The rigor of our design and evaluation determines whether patients experience genuine improvements in outcomes. By leading the demand for methodologically diverse, context-sensitive evidence, the Asia–Pacific oncology nursing community can bridge the gap from promising innovation to safe, scalable clinical improvement.

## CRediT authorship contribution statement

Karthik Adapa: Conceptualization, writing, reviewing, and editing. Suguna Kotte: Writing, reviewing and editing. Stefan Alexis Escobar Agreda: Writing, reviewing and editing. Mounika Metta: Writing, reviewing and editing. Resham Sethi: Reviewing and editing. Girish Patil: Reviewing and editing. Lukasz Mazur: Reviewing, editing and supervision. All authors have read and approved the final manuscript.

## Ethics statement

Not required.

## Data availability statement

Data availability does not apply to this article as no new data were created or analyzed in this study.

## Declaration of generative AI and AI-assisted technologies in the writing process

During the preparation of this work, the author(s) used ChatGPT and Claude to proofread and quality check for language/grammar. After using this tool/service, the authors reviewed and edited the content as needed and take full responsibility for the content of the publication.

## Funding

This study received no external funding.

## Declaration of competing interest

The authors declare no conflicts of interest. The views expressed in this article are solely those of the authors. In particular, the views of the corresponding author, Dr. Karthik Adapa, are his own and do not reflect the official positions of the World Health Organization (WHO), or the Division of Health care Engineering, Department of Radiation Oncology, School of Medicine at UNC Chapel Hill. The article has undergone the journal's standard publication procedures.

## References

[1] Burden J., Weerts H., Raz M. (2025). Paradigms of AI evaluation: mapping the landscape of evaluative frameworks and approaches. arXiv.

[2] Nagendran M., Chen Y., Lovejoy C.A. (2020). Artificial intelligence versus clinicians: systematic review of design, reporting standards, and claims of deep learning studies. BMJ.

[3] Vasey B., Nagendran M., Campbell B. (2022). Reporting guideline for the early-stage clinical evaluation of decision support systems driven by artificial intelligence: DECIDE-AI. Nat Med.

[4] Jabbour S., Chang T., Das Antar A. (2025).

[5] Myllyaho L., Raatikainen M., Mannisto T., Mikkonen T., Nurminen J.K. (2021). Systematic literature review of validation methods for AI systems. J Syst Softw.

[6] Reddy S., Rogers W., Makinen V.P., Coiera E., Brown P., Wenzel M., Weicken E., Ansari S., Mathur P., Casey A., Kelly B. (2021). Evaluation framework to guide implementation of AI systems into healthcare settings. BMJ health & care informatics.

[7] Sanchez-Martinez S., Camara O., Piella G. (2022). Machine learning for clinical decision-making: challenges and opportunities in cardiovascular imaging. Front Cardiovasc Med.

[8] Han R., Acosta J.N., Shakeri Z., Ioannidis J.P.A., Topol E.J., Rajpurkar P. (2024 May). Randomised controlled trials evaluating artificial intelligence in clinical practice: a scoping review. Lancet Digit Health.

[9] Iqbal D., Sam C., Attaullah A., Audrey L., J-PAL. AI Evidence Playbook (2026).

[10] Case Study 20 (2024). The $4 Billion AI Failure of IBM Watson for Oncology - Henrico Dolfing [Internet]. https://www.henricodolfing.ch/case-study-20-the-4-billion-ai-failure-of-ibm-watson-for-oncology/.

[11] Binns C., Low W.Y. (2024). Cancer: An Increasing Public Health Challenge in the Asia Pacific. Asia Pacific Journal of Public Health.

[12] Artificial Intelligence (AI (2026). Oncology Market Size, report by 2035.

[13] Thind B.S., Tsao C.K. (2025 Nov 4). Artificial intelligence in oncology: promise, peril, and the future of patient-physician interaction. Front Digit Health.

[14] Park S.H., Choi J.I., Fournier L., Vasey B. (2022). Randomized clinical trials of artificial intelligence in medicine: why, when, and how?. Korean J Radiol.

[15] Plana D., Shung D.L., Grimshaw A.A., Saraf A., Sung J.J.Y., Kann B.H. (2022 Sep 1). Randomized Clinical Trials of Machine Learning Interventions in Health Care: A Systematic Review. JAMA Netw Open.

[16] van de Sande D., Chung E.F.F., Oosterhoff J., van Bommel J., Gommers D., van Genderen M.E. (2024). To warrant clinical adoption AI models require a multi-faceted implementation evaluation. NPJ Digit Med.

[17] Jin M.F., Noseworthy P.A., Yao X. (2024 Aug 6). Assessing Artificial Intelligence Solution Effectiveness: The Role of Pragmatic Trials. Mayo Clin Proc Digit Health.

[18] Afshar M., Resnik F., Baumann M.R. (2025). A novel playbook for pragmatic trial operations to monitor and evaluate ambient artificial intelligence in clinical practice. NEJM AI.

[19] Curran G.M., Bauer M., Mittman B., Pyne J.M., Stetler C. (2012). Effectiveness-implementation hybrid designs: combining elements of clinical effectiveness and implementation research to enhance public health impact. Med Care.

[20] El Arab R.A., Abu-Mahfouz M.S., Abuadas F.H. (2025). Bridging the gap: from AI success in clinical trials to real- world healthcare implementation—a narrative review. Healthcare (Basel).

[21] Yang S., Gamalo M., Fu H. (2025). Integrating RCTs, RWD, AI/ML and statistics: next-generation evidence synthesis. arXiv.

[22] Di Bidino R., Daugbjerg S., Papavero S.C., Haraldsen I.H., Cicchetti A., Sacchini D. (2024 Nov 21). Health technology assessment framework for artificial intelligence-based technologies. Int J Technol Assess Health Care.

[23] Di Bidino R., Fasterholdt I., Daugbjerg S., Cicchetti A. (2026). Unlocking AI’s value: the HTA approach to responsible healthcare integration. BMJ Digit Health AI.

[24] Haverinen J., Turpeinen M., Falkenbach P., Reponen J. (2022). Implementation of a new Digi-HTA process for digital health technologies in Finland. International Journal of Technology Assessment in Health Care.

[25] Unsworth H., Dillon B., Collinson L. (2021). The NICE Evidence Standards Framework for digital health and care technologies - Developing and maintaining an innovative evidence framework with global impact. Digit Health.

[26] Gates Foundation, Novo Nordisk Foundation, Wellcome (February 20, 2026).

[27] World Health Organization (2021).

[28] Tun H.M., Rahman H.A., Naing L., Malik O.A. (2025 Apr 15). Artificial intelligence utilization in cancer screening program across ASEAN: a scoping review. BMC Cancer.

[29] Rivera S.C., Liu X., Hughes S.E. (2023). Embedding patient-reported outcomes at the heart of artificial intelligence health-care technologies. Lancet Digit Health.

[30] Concato J., Corrigan-Curay J. (2022). Hybrid clinical trials to generate real-world evidence: design considerations from a sponsor’s perspective. Contemp Clin Trials.

[31] Rosenthal J.T., Beecy A., Sabuncu M.R. (2025). Rethinking clinical trials for medical AI with dynamic deployments of adaptive systems. npj Digit Med.

